# Associations between EEG power and coherence and cognitive and language development across the first months of life

**DOI:** 10.21203/rs.3.rs-3178892/v1

**Published:** 2023-07-31

**Authors:** Ran Xiao, Beth Smith, Holly Bradley

**Affiliations:** Emory University; Children’s Hospital Los Angeles; Children’s Hospital Los Angeles

## Abstract

The neural processes underpinning cognition and language development in infancy are of great interest. We investigated EEG power and coherence in infancy, as a reflection of underlying cortical function of single brain region and cross-region connectivity, and their relations to cognition and language development. EEG recordings were longitudinally collected from 21 infants with typical development between 1 and 7 months. We investigated relative band power at theta (3–6Hz) and alpha (6–9Hz) and EEG coherence of these frequency bands at 25 electrode pairs that cover key brain regions. A correlation analysis was performed to assess the relationship between EEG measurements (frequency bands and brain regions) and raw Bayley cognitive and language developmental scores. In the first months of life, relative band power is not correlated with changes in cognitive and language scales. However, theta coherence is negatively correlated with receptive language scores between frontoparietal regions, and alpha coherence is negatively correlated with expressive language scores between frontoparietal regions. The results from this preliminary study are the first steps in identifying potential biomarkers of early cognitive and language development. In future work, we will confirm norm references of early cognitive and language development that can be compared with infants at risk for neurodevelopmental disabilities.

## 1. Introduction

Investigating neural development in the first months of life is critical for understanding the foundations upon which future higher level cognitive and linguistic abilities are built. By knowing more about this period, we can gain valuable insights into infant neural maturation, informing our understanding of typical and atypical childhood development. During infancy, the human brain undergoes substantial changes (Gibb & Kolb, 2017). Although efforts have been made to investigate and learn more about these changes, there is still much to be understood about brain development, specifically with regard to human cognition in the first months of life. In this period, cognition encompasses a wide range of skills, including language (Perszyk & Waxman, 2018). Although cognition and language are interconnected and involve some of the same processes (namely attention, working memory, and processing speed), language is a unique skill in that it requires the knowledge and development of syntax, morphology, and phonology (Deldar et al., 2020). Further, the necessity of entrenchment in social situations to acquire language sets it apart from other cognitive processes. The neural processes underpinning early linguistic and cognitive abilities are of increasing interest and yet are still relatively understudied in the very first months of life.

In this study, we investigated the correlations between resting-state (RS) electroencephalography (EEG) power and coherence with cognitive and language development longitudinally in infants 1–7 months of age. Cognitive and language developmental status were assessed using the Bayley Scales of Infant and Toddler Development (3rd edition) raw cognitive, receptive language, and expressive language subscale scores. We explored both theta and alpha frequency bands in our analysis, as per previous research signifying the importance of these bands in broad cognitive development (Cellier et al., 2021; Jones et al., 2020). EEG power and coherence are two commonly used measures in EEG research; EEG power is representative of activity in different frequency bands of the signal whereas coherence between electrodes shows present connections between neural regions (Nunez & Srinivasan, 2004). Using both measures together allows for a more comprehensive understanding of the cortical function underlying cognitive development. Further, RS EEG recordings provide an assessment of ongoing brain activity while an individual is awake, and so are appropriate to use to investigate cortical function development in infancy. It has been noted that most of the infant RS EEG literature has solely focused on infant alpha, and how this relates to learning, cognition, and developmental outcomes, but that further inquiry into RS EEG would benefit from “investigating associations between other cortical rhythms and cognitive development” (Anderson & Perone, 2018).

It is widely accepted that alpha band power is linked to basic cognitive processing (Klimesch, 2012). In infancy, RS EEG is associated with cognition and working memory, in a Piagetian A-Not-B task 8-month-old infants showed an increase in alpha coherence observed in frontal, parietal and occipital areas relative to baseline values (Bell, 2001). Also, RS frontal alpha at 10 months has been shown to be predictive of executive function at 4 years of age (Kraybill & Bell, 2013). Theta band power has also been linked to emotional and cognitive functioning skills in the second half of the first year of life, including attention modulation (Orekhova et al., 1999). Further, frontal theta and social attention and executive control skills have been linked in 5-month-old infants (Bazhenova et al., 2007), and frontal theta power in 12-month-old infants has been used to predict language and cognitive skills at 2, 3, and 7 years of age (Jones et al., 2020). Infants also show an increase from a baseline condition in theta power during events associated with learning or working memory (Orekhova et al., 2006). Perone & Gartstein (2019), using a parent-report of infant behavioral tendencies at 6 to 12 months old, found that lower levels of theta in frontal areas during baseline were associated with infant attention; namely, using another person to self-regulate. These findings provide evidence that theta and alpha band powers, specifically over the frontal cortex, are associated with cognitive and language skill development in later infancy (after 5 months of age).

The literature on functional connectivity of typically developing infant EEG that reflects the dynamic interactions and coordination of neural activities of different brain regions in younger infants is relatively sparse (Perone & Gartstein, 2019b); Whedon et al., (2016) found that a greater change in left frontal alpha EEG coherence between 5 and 10 months of age was positively associated with a multitude of cognitive abilities including receptive language, attentional control, and behavioral inhibition. Increased alpha coherence has been observed between 5 and 10 months of age alongside improvements in working memory performance (Cuevas et al., 2012; Cuevas & Bell, 2011). Further, in infants from 7 to 12 months of age, it has been found that increased performance on an A-not-B-task was associated with an increase in anterior/posterior coherence (Bell & Fox, 1992).

Our goal in the present study was to complement previous studies investigating EEG power and coherence in infancy and its relation to cognition and language, while expanding on these by assessing a novel group (younger infants longitudinally over the first half year of life). We did this via monthly data collections of RS EEG and Bayley Scales of Infant and Toddler Development (3rd edition) cognitive, receptive language, and expressive language subscale scores in infants aged 1–7 months of age. This work has the potential to identify early neural biomarkers of human cognition and language in the first half year of life. This is important because it can allow us to establish early brain function related to cognition and language that can be used to describe the developmental trajectories of language and cognition, and to compare with infants at risk for neurodevelopmental disabilities in future studies.

## 2. Methodology

### 2.1 Participants

EEG recordings (53 sessions) were longitudinally collected from infants with typical development (TD) (n=21; mean age = 4.23, SD = 1.51 months at the first visit). All infants were between 38 and 203 days of age, from singleton, full-term (38 weeks minimum gestation) births, had experienced no complications during birth, and had no known visual, neurologic, or orthopedic impairment. Further, no infant scored at or below the 5th percentile overall for their age on the Bayley Scales of Infant and Toddler Development (3rd edition). Both EEG data and Bayley scores were collected in monthly increments; 3–5 sessions were acquired for 19 participants, and 1 session was acquired for 2 participants.

### 2.2 Procedures

The procedures for this study have previously been described in full (Xiao et al., 2018). The study was approved by the Institutional Review Board of the University of Southern California, and a parent or legal guardian provided informed consent before participation. All methods were performed in accordance with the relevant guidelines and regulations. EEG data were acquired using a 32-electrode cap and Biosemi system at a sampling rate of 512 Hz. The data collection sessions are described as follows. First, 2 trials of 20 seconds resting-state EEG were recorded (researchers were encouraged to record 1 minute of baseline EEG here if the infant was cooperative). For the baseline condition, a glowing and spinning globe toy was presented out of reach to the infant to maintain attention and minimize movement. Second, the infants participated in the reaching condition in which they were presented with an interesting toy at midline. Third, the toy was removed for the non-reaching condition. Reaching and non-reaching trials were alternated 5 times. Finally, the baseline condition was repeated. In addition, at each visit, the Bayley Scales of Infant and Toddler Development (3rd edition) was administered to the infants to measure their language, motor, and cognitive development. Of these subscores, we used the raw cognitive score (RC) as the measure of cognitive developmental status, and receptive and expressive language scores (RRL and REL) as measures for language developmental status in the study. This present study is part of a larger project investigating the development of neuro-motor control during the first year of life. Other data, such as wearable motion sensor data and anthropometric data, were also recorded but were not analyzed in this study.

### 2.3 Preprocessing of EEG data

Infant EEG data is susceptible to external noise and artifacts, so a series of preprocessing techniques were applied to enhance the quality of the EEG signal. All preprocessing steps were performed by using the EEGLAB toolbox (ver. 13_6_5_b) (Delorme & Makeig, 2004). The data from all electrodes was first re-referenced to the average of T7 and T8, then a 0.3–30Hz bandpass infinite impulse response (IIR) filter was applied to the data. The EEG baseline conditions were extracted from the EEG recordings and visually inspected; any large fluctuations were removed. Kurtosis indices were then calculated for all electrodes, if any electrode had a Kurtosis index falling beyond 5 standard deviations of all electrodes, then it was rejected, and its signal was interpolated by surrounding electrodes. A common average reference was applied by re-referencing each electrode to the average of all electrodes to filter out common-mode artifacts. Finally, an independent component analysis was conducted to separate the baseline EEG signal into independent components (ICs) originating from the brain source and unwanted artifacts (Delorme et al., 2012). Any components caused by electrocardiography, lateral eye movements, eye blinks, and motion artifacts were visually identified and removed to enhance the signal quality for subsequent analysis. To ensure the maximum retention of information related to brain activities, each IC was evaluated based on its temporal, spectral, and spatial features. This resulted in the exclusion of 2 to 3 ICs for most sessions.

### 2.4 Spectral Analysis of EEG

Power spectral densities (PSD) were estimated on the preprocessed EEG data using Welch’s method (Welch, 1967). The “pwelch” function in MATLAB (MathWorks Inc., Natick, MA, USA) was used for the PSD estimation. A 2-second window length Hann window was chosen for the PSD estimation, with a 50% overlap between segments, resulting in a 0.5 Hz frequency resolution for capturing the spectral activity changes in the infant EEG data.

**Figure 1:**
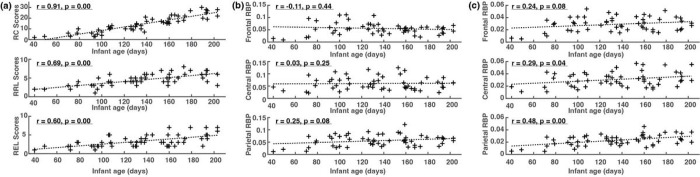
(a) Correlation between three Bayley raw subscale scores and infant age in days; (b) Correlation between theta relative band powers (RBP) and infant age in days; (c) Correlation between alpha RBPs and infant age in days. RC = Bayley raw cognitive subscale score; RRL = Bailey raw receptive language subscale score; REL = Bayley raw expressive language subscale score; RBP = relative band power.

To account for variation across sessions and ages and allow for comparison across all spectral activities from individual sessions, PSDs were transformed into relative powers (between 0 and 30 Hz). For each frequency bin within this range and each electrode, relative power was computed by dividing PSD by the sum PSD from all bins. This transformation adjusted the PSDs into energy ratios within a sub-30 Hz frequency range to allow cross-session comparisons to be made. Theta and alpha relative band powers (RBP) were computed for each session by adding together all relative powers of all frequency bins within 3–6 Hz and 6–9 Hz, respectively. Further, theta and alpha rhythmic activities from frontal, central, and parietal brain areas were calculated by averaging key representative electrodes measuring activities in these brain regions (frontal: F3, Fz, and F4; central: C3, Cz, and C4; parietal: P3, Pz, and P4). We first investigated the correlation between RBPs of two frequency bands and infant age (in days), and the correlation between the three Bayley subscale scores and infant age (in days). Based on the obtained results (see Fig. 1), we determined the infant age as a confounding factor for discovering EEG biomarkers that are strongly associated with cognitive and language development. Therefore, the partial correlation analysis was performed between RBPs at two frequency bands (i.e., theta and alpha) at those three brain regions and each of the three Bayley subscales (i.e., RC, RRL, and REL), controlling for the effect of infant age. In total, there were 18 combinations entered into the analysis to identify potential markers in EEG relative band powers associated with cognitive and language skill development. Additional procedures were implemented to adjust for multiple comparisons and investigate the impact of repeated measures in the data, which were described in detail in Section 2.6 below.

### 2.5. Connectivity Analysis of EEG

To investigate the interaction and coordination of neural activities across different brain regions, we evaluated the functional connectivity by computing the magnitude-squared coherences of theta and alpha bands for 25 pairs of electrodes covering frontal, central, parietal, and occipital cortices. These electrode pairs were selected to capture both within-region and cross-region connectivity, as well as ipsilateral and bilateral coordination. The full list of 25 electrode pairs can be found in Fig. 3. The magnitude-squared coherence was calculated by first estimating both single-electrode (i.e., auto-spectral density) and cross-electrode PSD (i.e., cross-spectral density) for each electrode pair by following the same parameters as the previous analysis, i.e., a 2-second Hann window with 50% overlap based on Welch’s method. Then, the coherence was achieved by dividing the square of cross-spectral density by the multiplication of auto-spectral density from each electrode in the electrode pair. The coherence calculation was performed for all electrode pairs using the MATLAB function “mscohere”.

To study developmental changes in brain connectivity, we evaluated mean coherences in the theta and alpha bands from each of these 25 electrode pairs across the raw Bayley cognitive, receptive language, and expressive language subscale scores. Again, the partial Pearson’s correlation was calculated for each combination of connectivity measures and Bayley subscale scores with infant age as the confounding effect. There were in total 150 combinations entered into the correlation analysis, as shown in Fig. 3. Same to the spectral analysis, additional procedures were implemented to adjust for multiple comparisons and investigate the impact of repeated measures in the data, which were described in detail in the section below.

### 2.6. Statistical Analysis

To test the statistical significance of the correlations in the partial correlation analyses, the t-score was firstly calculated by

Eq. (1) $$t=r*\sqrt[{2}]{\frac{n-2}{1-r^{2}}}$$

where r is the sample linear partial correlation coefficient controlling for age and n is the sample size in the correlation analysis (n=53). Next, the p-value was calculated by comparing the t-score against the t-distribution in a two-tailed test with the null hypothesis that there is no linear correlation between the two variables in the correlation analysis. The significant level alpha was set as 0.05. In all tests in the study, p values were adjusted by the Benjamini-Hochberg procedure for multiple comparisons.

Given that multiple sessions were available from different participants, there existed the issue of repeated measures for analysis in the study data that reduced the effectiveness of modeling with simple linear regression. So, a linear mixed-effects model (LMM) was adopted to account for the non-independency of data arising from participants. The LMM added random effect terms to the model to tackle non-independency, resulting in more accurate representations of outcomes (Krueger & Tian, 2004). The LMM models were designed as follows in Wilkinson notation:

Eq. (2) $${Var}_{\mathrm{Resp}}=1+{\mathrm{Var}}_{\mathrm{Pred}}+\mathrm{Age}+(1|\mathrm{Participants})+\epsilon$$

Where ${\mathrm{Var}}_{\mathrm{Pred}}$ is the predictor variable, which includes relative band powers and coherences in theta and alpha bands in the present study, with Age also included as a predictor to control for its confounding effect in the LMM analysis. ${Var}_{\mathrm{Resp}}$ is the response variable, which includes the three Bayley subscale scores (i.e., RC, RRL, and REL). The models capture changes in each of the responsible variables across each of those predictor variables by considering both fixed and random effects for the slope and intercept terms. The first two elements on the right side of Eq. (2) denote the fixed-effect terms, including fixed-effect intercept and slope from the EEG biomarkers and age as predictors; the term (1|Participants) specifies the random effects of the model that are imposed by the grouping factor, i.e., participants; ε is the error term. With the LMM analysis, we investigated whether there were still strong associations between the targeted predictors (i.e., EEG biomarkers) and different cognitive and language developmental scores, even after controlling for the effect of age and accounting for repeated measures from participants in the linear mixed-effects model.

## 3. Results

### 3.1 Changes in Bayley subscale scores and EEG relative band powers along with maturation

Figure 1(a) shows significant and positive correlations between the three cognitive scores (RC, RRL, REL) and infant age in days, illustrating the development of key cognitive and language skills along with maturation. Raw cognitive scores showed a strong positive correlation with age r=0.91,p<0.01, raw receptive language scores showed a moderate positive correlation with age r=0.69,p<0.01, and raw expressive language scores showed a moderate positive correlation with age r=0.60,p<0.01. Compared to those Bayley subscales, EEG relative band powers present weaker correlations with age (see Fig. 1(c)), but still reached statistical significance from alpha RBPs measured from central (r=0.29,p<0.05) and parietal lobes (r=0.48,p<0.01).

### 3.2 EEG spectral power changes along with cognitive development

**Figure 2:**
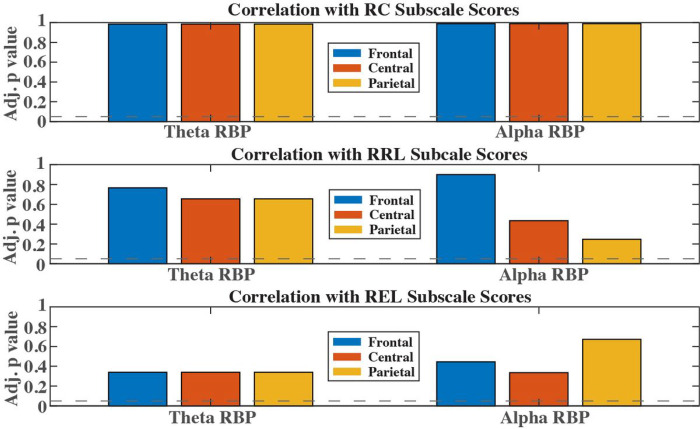
Adjusted p values for Pearson’s correlation between three cognitive scores and theta and alpha band relative powers. Horizontal lines denote the significant level at 0.05.

Figure 2 shows the adjusted p values (after correction for multiple comparisons) for theta and alpha relative band power correlating with RC, RRL, and REL in frontal, central, and parietal brain regions. After correction for multiple comparisons, and accounting for the confounding effect of age, none of the RBPs from any frequency bands or brain regions reached statistical significance (p>0.05), failing to reject the null hypothesis that there is no significant correlation between EEG RBPs in the theta and alpha bands and the three Bayley subscale scores.

### 3.4. Brain connectivity changes along with cognitive and language development

**Figure 3:**
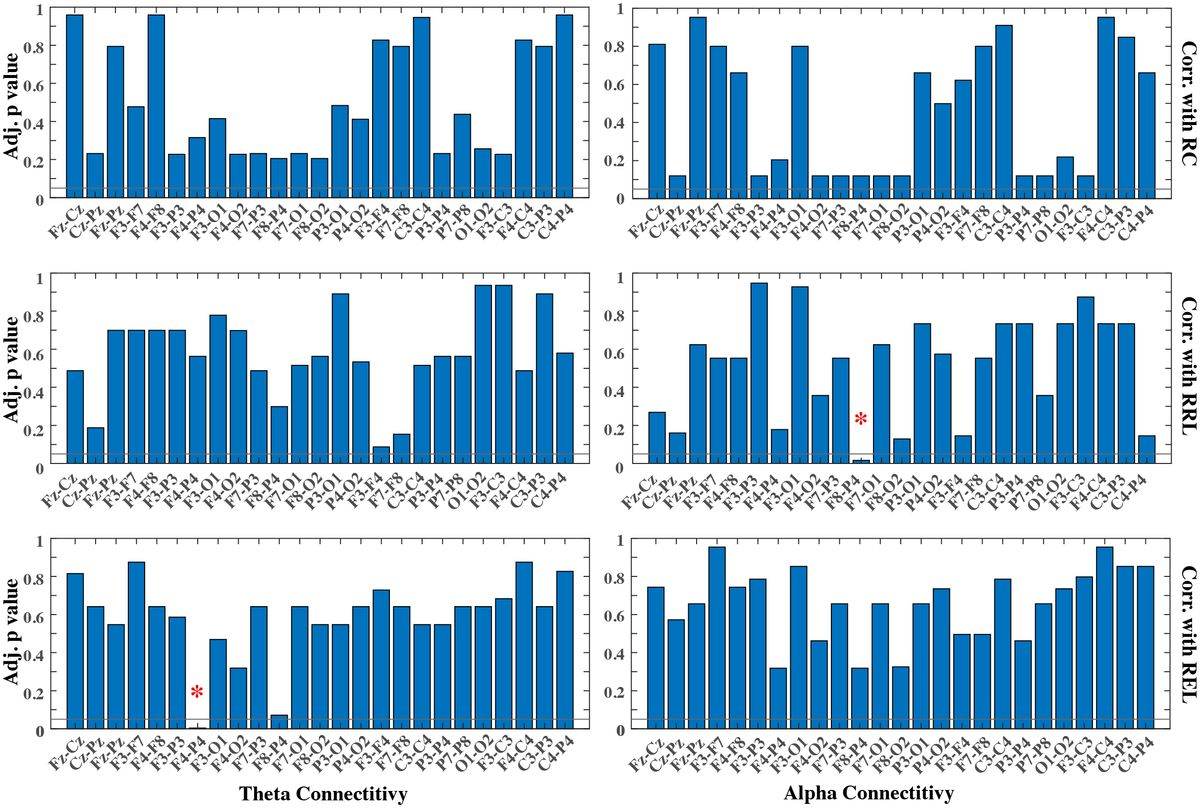
Adjusted p values for correlations between cross-electrode connectivity and three Bayley subscale scores. Each row presents the partial correlation results for one subscale (RC, RRL, and REL). Each column presents results using each frequency band (theta and alpha) for deriving the connectivity. Horizontal lines denote the significant level at 0.05. * indicates statistically significant correlation after adjustment for multiple pair comparisons and accounting for age as the confounding factor. RC = Bayley raw cognitive subscale score; RRL = Bayley raw receptive language subscale score; REL = Bayley raw expressive language subscale score.

**Figure 4:**
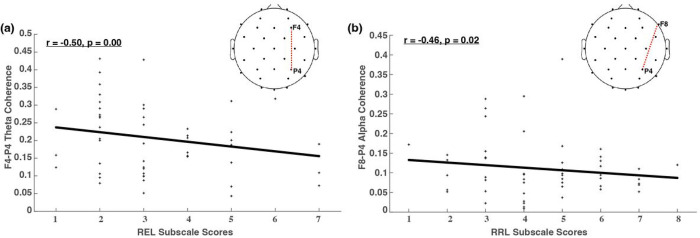
Brain regions with significant connectivity changes along Bayley language subscale scores.

Figure 3 illustrates the adjusted p values (corrected for multiple comparisons) across all electrode pairs for theta and alpha coherences. Out of the 25 electrode pairs evaluated in this present study, one pair (F4-P4) shows significant changes in theta connectivity with respect to REL. In terms of alpha coherence, one pair (F8-P4) shows significant changes in alpha connectivity with regard to RRL. All correlations are negative, illustrating decreased connectivity between frontal and parietal regions as performance increases on receptive and expressive language skills assessed by the Bayley. These findings reveal unique frontoparietal connectivity changes across language development for both theta and alpha coherences. Figure 4 shows the brain regions with significant connectivity changes along three Bayley scores, and topographies highlighting the relationships between brain regions with significant connectivity along two of the three Bayley subscale scores (i.e., RRL and REL). It reveals negative correlations in both theta (r=−0.45,p<0.01) and alpha (r=−0.46,p<0.01) connectivity with Bayley language subscales. And the correlations both stem from connectivity between the frontal and parietal lobes.

Table 1 presents the results of the LMM analysis, examining the associations between F4-P4 theta connectivity $\left({Coh}_{\text{Theta:F4-P4}}\right)$ and REL subscale scores, as well as between F8-P4 alpha connectivity $\left({Coh}_{\text{Alpha:F8-P4}}\right)$ and RRL subscale scores. The analysis demonstrates robust and statistically significant associations between both EEG connectivity-based markers and Bayley language subscale scores. These associations remained significant after adjusting for potential confounding factors such as infant age and accounting for repeated measures from multiple sessions involving the same participants in the study data.

## 4. Discussion

This paper is the first to investigate EEG theta and alpha power and coherence longitudinally in the first half year of life using a standardized developmental assessment for cognitive and language development. While we found no significant contributions of alpha and theta band powers as predictors to the changes in cognitive and language developmental scale scores, we did find connectivity changes in these frequency bands were associated with language development. Specifically, we found that theta coherence is negatively correlated with receptive language scores between frontoparietal regions and that alpha coherence is negatively correlated with expressive language scores between frontoparietal regions. The present study complements existing work in this field by demonstrating that, in the first months of life, language development (both receptive and expressive) is associated with frontoparietal interhemispheric theta and alpha band connectivity changes.

We did not find any significant correlations between alpha and theta EEG RBPs and the three Bayley subscale scores. We expected to see infant alpha band power associated with cognition as it has been shown to relate to basic cognitive processing at later ages. Most previous research reported an increase in frontal alpha associated with a cognitive task in comparison to a baseline (Klimesch, 2012; Kraybill & Bell, 2013); however, Bell (2001) found this in frontal, parietal and occipital areas. Some research has found that alpha oscillations in the parietal lobe are associated with attention (Orekhova et al., 2001); for example, infants aged 8–11 months of age show attenuated alpha PSD over posterior brain regions as they watch a tv show in comparison to being in total darkness. Further, in adults, it has been found that parietal alpha power is reflective of inhibition effects in the parietal attention network (Benedek et al., 2014). This present study failed to find these associations when accounting for the confounding effects of infant age, indicating that alpha band power and its association with cognitive development might emerge at later ages. This corroborates with our previous findings and other studies that indicate prominent alpha band power starts to emerge at least 6 months of age. It is also possible that high variability within and between individuals early in infancy makes it difficult to find associations. Future work should continue to explore the association between alpha band power and cognition longitudinally in the developing infant over the first year of life to investigate if relative band power may be associated with the ongoing underlying development of cognition at a certain stage in infancy. As cognitive and language abilities develop and neural functions are established, neural processing may shift towards other frequency bands or regions. Future research should also aim to investigate other frequency bands, such as beta and delta, to assess this.

In terms of coherence, we found that alpha was significantly correlated with raw expressive language scores in frontoparietal regions. Alpha coherence has been used as a measure of cognitive and language function in adults (Stam et al., 2006) and individual differences in alpha coherence in infancy have been shown to be indicative of later cognitive outcomes (Cuevas et al., 2012; Cuevas & Bell, 2011). All previous studies have investigated the development of cognition and language in infants older than 5 months of age; this present study illustrates that alpha coherence is related to infant expressive language development in the first half year of life. Specifically, across this time period in life, increased expressive language skills are correlated with decreased frontoparietal connectivity. Further, we also found that theta was significantly correlated with receptive language in frontoparietal regions, demonstrating the same relationship of decreasing connectivity with improved receptive language performance. Together, these findings demonstrate alterations in inter-hemisphere frontoparietal theta connectivity with receptive language development, and inter-hemisphere frontoparietal alpha connectivity with expressive language development. One possible explanation for this could be a greater efficiency in neural processing leading to a decrease in the synchronization of neural activity, reflecting the development of more focused and targeted neural processing as language and cognition functions develop. It is also possible that the decreases found in connectivity as skills develop could reflect a normal maturation process in the developing brain as it undergoes reorganization. More research is needed to fully understand the decrease in frontoparietal theta and alpha connectivity as language develops over the first half year of life.

As previously mentioned, investigating the underlying cortical function of language and cognition development across the first months of life, as in this study, can provide important insights into typical developmental trajectories that can be compared with infants at risk for neurodevelopmental disabilities in future studies. Our results seem to show that cognition, receptive language, and expressive language are not independent facets of development in the first months of life, but receptive language is associated with theta coherence, and expressive language is associated with alpha coherence. Language and cognition are both complex constructs involving multiple neural processes, and future work should continue to disentangle the underlying neural mechanisms of these two domains across the first months of life. Limitations exist with regard to this current study. This present study was a preliminary one, with only a small number of infants who were tested at varying ages over an unequal number of sessions. To clarify the existence of these potential biomarkers of cognitive and language maturation, we will use our results to design adequately powered studies in the future. Further, some issues exist in that it is difficult to collect true RS EEG in infants. RS EEG studies differ quite drastically in methodology (Anderson & Perone, 2018) with regard to condition, analysis, and brain region of interest among others which may influence the data. Infant RS EEG often involves engaging the child in a task that will keep them calm while minimizing eye and motor movements. These tasks differ from study to study but tend to involve the infant playing (Benasich et al., 2008), watching a video on a screen (Perone & Gartstein, 2019a), or watching a spinning globe toy, as in this study. It has been argued that the child brain does not rest in a manner that is consistent with adult brains (Camacho et al., 2020), and that rest periods in infancy may actually represent periods of increased cognitive control. Future work should aim to adopt a standardized baseline EEG measure so that RS data can be more easily compared across groups and establish consistencies about what infant RS EEG really is, and how it is best measured.

To summarize, this work is the first step in identifying potential biomarkers of cognitive and receptive and expressive language development during RS EEG across the first months of life. For the next steps, we will use these preliminary results to design an adequately powered study to further investigate the relationships identified here across the first year of life. This will allow us to confirm norm references of early cognitive and language development. We can use these norm references in future work to compare with infants at risk for neurodevelopmental disabilities. The ability to identify atypical cognitive and language development in the first months of life would support early, targeted interventions and optimal developmental outcomes.

## Figures and Tables

**Table 1:** Linear mixed-effect model analysis for evaluating the associations between cross-region brain connectivity and Bayley language subscales.

LMM model	Name	t-statistics	Degree of freedom	p-value
REL = 1 + Coh_Theta:F4–P4_ + Age + (1|Participants) + *ϵ*	Intercept	1.86	50	0.07
	Coh_Theta:F4–P4_	−4.23	50	**0.00** *
	Age	7.24	50	0.00*
RRL = 1 + Coh_Alpha:F8–P4_ + Age + (1|Participants) + *ϵ*	Intercept	2.49	50	0.02*
	Coh_Alpha:F8–P4_	−3.74	50	**0.00** *
	Age	8.41	50	0.00*

* indicates statistical significance.

## Data Availability

All relevant data are available from the Figshare repository at the following DOI: 10.6084/m9.figshare.5598814.v1.
